# The magnitude and determinants of delayed initiation of antenatal care among pregnant women in Gambia; evidence from Gambia demographic and health survey data

**DOI:** 10.1186/s12889-023-15506-0

**Published:** 2023-03-30

**Authors:** Solomon Gedlu Nigatu, Tilahun Yemanu Birhan

**Affiliations:** Department of Epidemiology and Biostatistics, Institute of Public Health, College of Medicine and Health Science, University of Gondar, Gondar, Ethiopia

**Keywords:** Antenatal care, Pregnant women, Late initiation and Gambia

## Abstract

**Background:**

Despite gains throughout the 20th century, maternal health remains a major public health concern. Despite global efforts to enhance access to maternal and child healthcare services, women in low- and middle-income countries still have a high risk of dying during pregnancy and after birth. This study aimed to determine the magnitude and determinants of late antenatal care initiation among reproductive age women in Gambia.

**Method:**

Secondary data analysis was conducted using the 2019-20 Gambian demographic and health survey data. All reproductive age women who gave birth in the five years preceding the survey and who had an antenatal care visit for the last child were included in this study. The total weighted sample size analyzed was 5310. Due to the hierarchical nature of demographic and health survey data, a multi-level logistic regression model was performed to identify the individual and community level factors associated with delayed first antenatal care initiation.

**Result:**

In this study, the prevalence of delayed initiation of initial antenatal care was 56% ranged from 56 to 59%. Women with age 25–34 [Adjusted Odds Ratio = 0.77; 95% CI 0.67–0.89], 35–49 [Adjusted Odds Ratio = 0.77; 95% CI 0.65–0.90] and women reside in urban area [Adjusted Odds Ratio = 0.59; 95% CI 0.47–0.75] respectively had lower odds of delayed first antenatal care initiation. While women with unplanned pregnancy [Adjusted Odds Ratio = 1.60; 95% CI 1.37–1.84], no health insurance [Adjusted Odds Ratio = 1.78; 95% CI 1.14–2.76] and previous history of cesarean delivery [Adjusted Odds Ratio = 1.50; 95% CI 1.10–2.07] had higher odds of delayed initiation of antenatal care.

**Conclusion:**

Despite the established advantages of early antenatal care initiation, this study revealed that late antenatal care initiation is still common in Gambia. Unplanned pregnancy, residence, health insurance, history of caesarian delivery, and age were significantly associated with delayed first antenatal care presentation. Therefore, focusing extra attention on these high-risk individuals could reduce delayed first antenatal care visit and this further minimizes maternal and fetal health concerns by recognizing and acting early.

## Introduction

Despite gains throughout the 20th century, maternal health remains a major public health concern [1, 2]. Globally, there were 152 maternal deaths for every 100,000 live births in 2020 [3, 4]. Despite the fact that the majority of maternal deaths are preventable, the majority (94%) found within limited resource settings [5]. Two-thirds of all maternal deaths worldwide occur in Sub-Saharan Africa (SSA) [6, 7]. While there have been international efforts to improve maternal and child health services, such as offering health education, developing health infrastructure, and screening sexually transmitted diseases for early detection and prevention of unfavorable pregnancy outcomes, women in low- and middle-income countries still face a high risk of death during pregnancy and after delivery [2, 8]. The use of antenatal care (ANC) services is a crucial indicator of how well the Sustainable Development Goals 3 (SDGs 3) are being achieved [9]. Reducing adverse pregnancy outcomes, such as maternal death and stillbirth, requires the early implementation and adequate use of ANC services [10, 11]. In addition to identifying risk factors, ANC exposes women to health education about danger signs and birth readiness and encourages them to give birth in a medical facility or with a skilled attendant [1, 10, 12–14]. Attending ANC during pregnancy allows pregnant women to gain knowledge that they can use during pregnancy and after giving birth [15–17]. The World Health Organization (WHO) and the Gambian Ministry of Health recommend eight antenatal care (ANC) visits for healthy pregnancies, with the first examination starting before 12 weeks of gestation [8, 18]. But studies show that the vast majority of women in sub-Saharan Africa begin antenatal care much later than recommended [11, 19–23]. Many pregnant women in West Africa, particularly teenage girls, start their antenatal care later than necessary, depriving them of the opportunity to receive preventive and curative services [2, 6, 22, 24–26]. Pregnancy outcomes and the number of prenatal visits or gestational age at ANC initiation have been related in epidemiological studies [17, 27]. Initiating ANC later than recommended may result in worse outcomes, such as low birth weight and preterm birth, and raise the overall cost of prenatal care [28–30]. According to earlier studies, ANC late onset initiation may have a greater impact on outcomes than visit frequency [16, 29, 31–33]. According to earlier research, women who initiate ANC later tend to be younger, more gravid or parous, single, of lower socioeconomic status, less educated, and have less access to healthcare services [24, 26, 34–36]. Women who experience unplanned pregnancies, find out they are pregnant later than average, and have had healthy pregnancies in the past are also more likely to start ANC later in their pregnancy [20, 30, 37–39]. Additionally, if women have had bad experiences in the past or have a negative perception of the quality of the services, they will not attend ANC [30, 40, 41]. The use of ANC has been reported to be influenced by cultural conceptions of pregnancy and beliefs, which may cause mothers to delay or skip ANC visits [41]. According to other studies, the reasons for the delayed initiation of ANC include younger ages, unplanned pregnancies, and ignorance of the value of early ANC [11, 30, 36, 42–44]. No study has been conducted at national level in Gambia to investigate the magnitude and its predictors of late ANC contact. Therefore, the current study analyzed a data from Gambia demographic and health survey (GDHS) to determine magnitude of late ANC initiation and to determine its predictors among women attending antenatal care service in Gambia.

## Methods and materials

### Study design, area, and period

The data set was accessed from the demographic and Health Survey (DHS) website (http://www.dhsprogram.com) after registering and stating the purpose of the study. The Gambia is the home of 1,692,865 people according to the 2013 national census done by the Gambia Bureau of Statistics [45]. It has a dense and multi-ethnic population. The administration is divided into eight Local Government Area (LGA). Those eight LGA are Banjul, Kanifing, Brikama, Mansakonko, Kerewan, Kuntaur, Janjanbureh and Basse. The health system of the country is a three-tier (primary, secondary, and tertiary level) [46]. The study period was from 21st November 2019 to 30th March 2020.

### Sample and study population

All women in the reproductive age group in the Gambia were the source population. The Source population was all women with a birth in the last 5 years before the survey. The birth should be the most recent birth if the woman had multiple births. Women who had birth history within the last 5 years before the survey but had no ANC recorded were excluded.

### Sample size and sampling procedure

Sample weighted was done to avoid over or under representative of each LGA then the final sample was 5310 women who had a birth history in the last 5 years before the survey and had recorded ANC visits. Sample selection is done using a multistage sample method. At the initial, the country was divided into eight LGA and this LGA was stratified into urban and rural. Using probability proportional 281 enumeration areas (EA) were selected from both urban and rural residencies. The next stage was selecting a household. A systematic random sampling method was employed to select a household and 7,025 households were selected. The detailed sampling procedure is also available on GDHS 2019-20 report [47].

### Dependent variable

The dependent variable was late ANC visit initiation of pregnant women. If Women’s first ANC visit happened after 16 weeks of gestational age, it was considered as a lately initiated ANC visit. GDHS reported the first ANC visit in months so that it was recorded as “1” when the gestational age was greater than four months. Otherwise, it was early initiation when it was booked within four months and recoded as “0”.

### Independent variables

The predictor variables for this study were individual and communality level variables. Among the individual variables some of the were socio-demographic variable: age of women, marital status, residency, ethnicity, women education, husband education, religion, women occupation, wealth index, media exposure, had health insurance, sex of household, household size, and distance from health facilities. The other individual-level variables were Obstetric-related variables such as ANC, parity, stillbirth, abortion history, planned pregnancy, and cesarean section History [27, 46, 48–50].

### Data collection procedure

The study was conducted based on GDHS data by accessing from the DHS program official database [51] after permission was granted through an online request by explaining the objective of our study. The raw data was collected from all parts of the country on childbearing aged women using a structured and pre-tested questionnaire. We used the Individual Record (IR file) data set and extracted the outcome and independent variables.

### Data management and analysis

The data were weighted using sampling weight, primary sampling unit, and strata before any statistical analysis to restore the representativeness of the survey and to tell the STATA to undertake in to account the sampling design when calculating standard errors to obtain reliable parameter estimates. Cross tabulation and summery statistics were conducted to describe the study population by STATA 16.

### Multi-level analysis

The outcome variable was a binary (late initiated ANC or early initiated ANC) and DHS used a multistage sampling method, women within the same cluster exhibited the same characteristics, to address this issue multilevel mixed-effect logistic regression model was the best. First, bivariable multilevel mixed-effects logistic regression analysis was done separately on both the individual-level and community-level variables to identify candidate variables for multivariable analysis and P-value < 0.2 was used as a cut point. In multivariable multilevel mixed-effects logistic regression analysis was done and a variable with a p-value ≤ 0.05 was declared as significant predictors of late initiated ANC visit.

### Model building

For the current study generally 4 models were fitted. The first was the null model (Model 0) (without predictor variable) used to check variation in community regarding late initiation of ANC and provide evidence to assess random effects at the community level. The second model (Model I) was the multivariable model adjustment for individual-level variables. The third model (Model II) was adjusted for community-level variables. The last model (Model II) all candidate variables from both individual and community-level variables were fitted with the outcome variable.

### Parameter estimation methods

The fixed effects were used to estimate the association between the likelihood of late ANC visit initiation and explanatory variables at both community and individual level and were expressed as odds ratio with 95% confidence interval. Regarding the measures of variation (random-effects) intra cluster correlation coefficient (ICC), Proportional Change in Community Variance (PCV) and median odds ratio (MOR) were used.

The MOR helps to translate the area level variance in the widely used odds ratio (OR) scale. The MOR is defined as the median value of the odds ratio between the area at the highest risk and the area at the lowest risk when randomly picking out two areas. The MOR can be understood as the increased risk that (in median) would have if moving to another area with a higher risk.

It is computed by; MOR = exp[√(2×Va)×0.6745] [52].

Where; VA is the area level variance, and 0.6745 is the 75th centile of the cumulative distribution function of the normal distribution with mean 0 and variance 1. Whereas the proportional change in variance is calculated as PCV = [(VA-VB)/ VA]*100 [53].

Where; where VA = variance of the initial model, and VB = variance of the model with more terms.

## Result

### Socio-demographic characteristics

Of the total, almost half of the study participants (n = 2,643; 49.8%), were aged between 35 and 34. Four thousand eight hundred seventy-seven (91.9%) of the studied women were married. Nearly two-thirds of the study subjects (n = 3,549; 66.8%) were urban by their residency. Almost all study participants (n = 5,176; 97.5%) were not had health insurance. Among the study participants (n = 3, 941; 74.2%) were had no problem accessing health facilities (Table 1).

**Table 1:** Background characteristics of study participant

Variable	Category	Frequency (N = 5310)	percent
Age	15–24	1,212	22.8
	25–34	2,643	49.8
	35–49	1,455	27.4
Marital status	Single	257	4.8
	Married	4,877	91.9
	Widowed	58	1.1
	Divorced	118	2.2
Residency	Urban	3,549	66.8
	Rural	1,761	33.2
Ethnicity	Mandinka/Jahanka	1,700	32.0
	Wollof	673	12.7
	Fula/TUkulur/Lorobo	1030	19.4
	Sarahule	415	7.8
	Non-Gambia	685	12.9
	Others	807	15.2
Local Government Area	Banjul	56	1.1
	Kanifing	969	18.2
	Brikama	2,185	41.1
	Mansakonko	226	4.3
	Kerewan	609	11.5
	Kuntaur	310	5.8
	Janjanbureh	335	6.3
	Basse	620	11.7
Women Education	No education	2,426	45.7
	Primary	933	17.6
	Secondary	1,699	32.0
	Higher	252	4.7
Husband Education	No education	2,400	49.4
	primary	730	15.0
	Secondary	1,334	27.4
	Higher	399	8.2
Religion	Muslim	5,163	97.2
	Christianity	147	2.8
Women occupation	Had work	3,537	66.6
	Not working	1,773	33.4
Wealth index	Poor	2,253	42.4
	Middle	1,115	21.0
	Rich	1,942	36.6
Media exposure	Yes	4,367	85.9
	No	718	14.1
Had health insurance	No	5,176	97.5
	Yes	134	2.5
Sex of household	Female	882	16.6
	Male	4,428	83.4
Household size	1–4	412	7.8
	5–90	4,898	92.2
Distance from health facilities	Not a big problem	3,941	74.2
	Big problem	1,369	25.8

### Obstetric-related characteristics

A high proportion of women (95.8%) had fewer than eight ANC visits during their last pregnancy time. Regarding stillbirth, 4767 (89.9%) of the participants had a history of stillbirth in their life. About four-fifths (79.7%) of the women who gave responses were planned to become pregnant (Table 2).

**Table 2:** Reproductive characteristics of pregnant women in Gambia

Variable	Category	Frequency (N = 5310)	percent
ANC	< 8	5,086	95.8
	>=8	224	4.2
Parity	Prim-pareous	1,066	20.1
	Multi- pareous	4,244	79.9
Still Birth	No	543	10.2
	Yes	4767	89.8
Abortion History	No	4,767	89.8
	Yes	543	10.2
Planned pregnancy	Yes	4,231	79.7
	No	1,079	20.3
CS History (5306)	Yes	221	4.2
	No	5,085	95.8

### The magnitude of late ANC initiation in Gambia

**Fig. 1:**
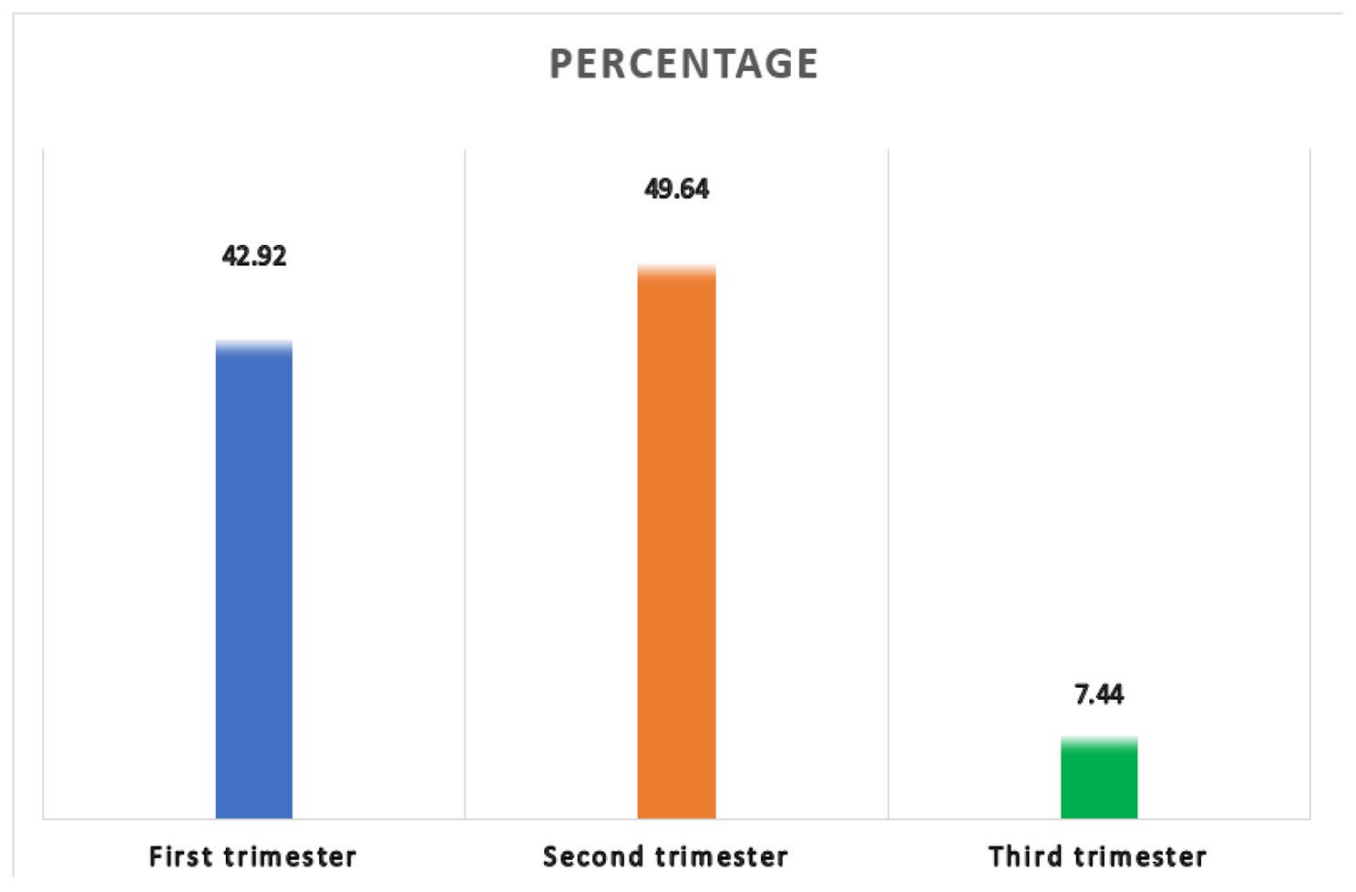
Prevalence of first ANC intiation with gestational age of reproductive women in Gambia

**Fig. 2:**
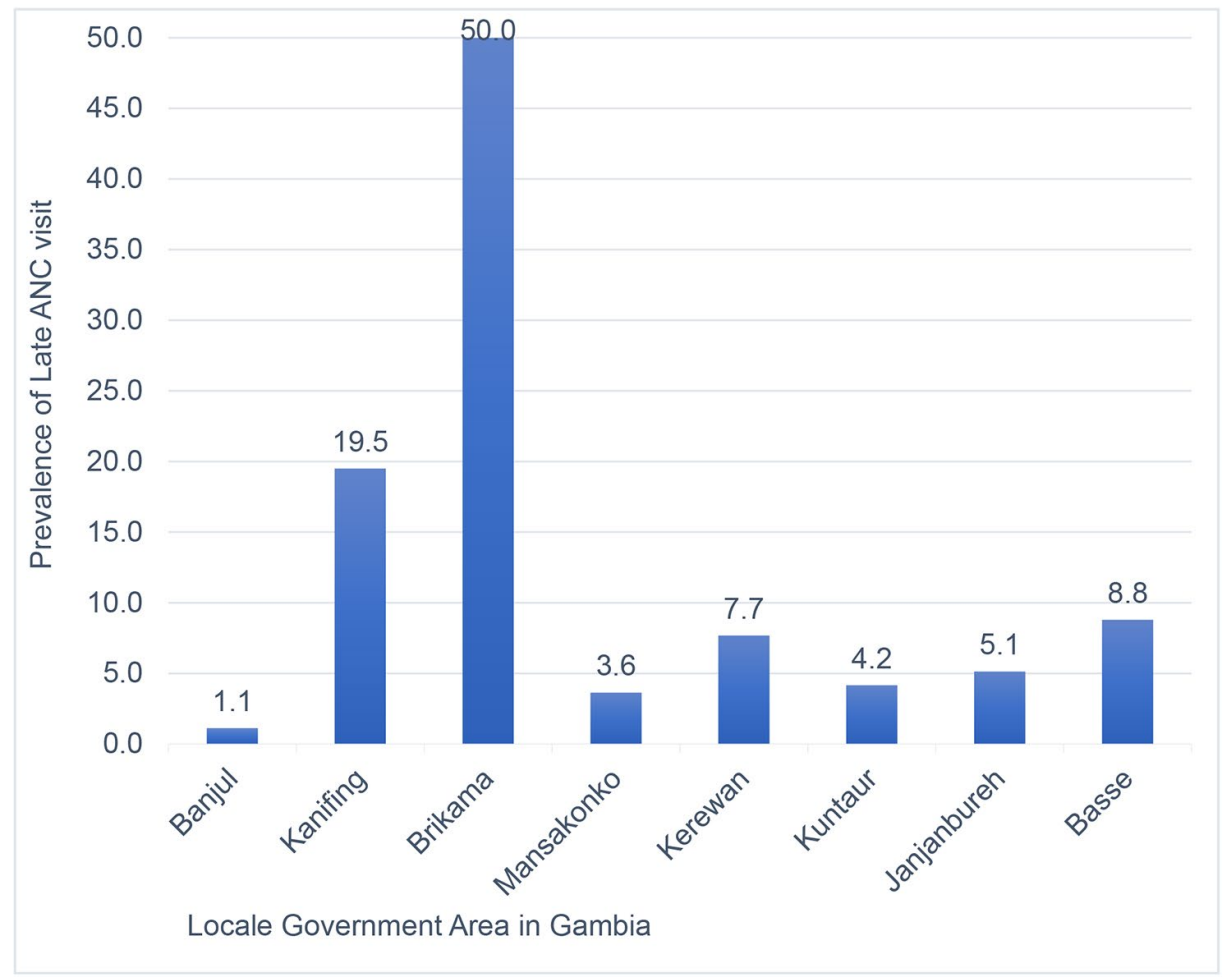
Prevalence of first ANC initiation at different local government area in Gambia

In this study, we found that the magnitude of late ANC initiation was 57.08 (95% CI: 55.75, 58.42). Of those who had delayed ANC initiation, the majority (49.64%) of study participants had their first ANC initiation at second trimester (Fig. 1).The highest prevalence of late ANC initiation recorded in Brikama local government area of Gambia. It consisted half of the total women who is lately initiated their ANC visit (Fig. 2).

#### Random effect analysis and model fitness

Table 3 revealed that in the null model, about 12% of the total variation on delayed initiation of first ANC visit was occurred the clustered level and it is attributable to the community level factors. In addition, the null model had the highest MOR value (2.17) indicating when randomly select an individual from one cluster with a higher risk of delayed initiation of first ANC booking and the other cluster at lower risk, Individuals at cluster with a higher risk of delayed initiation of first ANC presentation had 2.17 times higher odds of having a delayed first ANC initiation as compared with their counter parts. Furthermore, the highest (62%) PCV in the full model (Model III), indicates that 62% of the community-level variation on delayed first ANC initiation was explained by the combined factors at both the individual and community levels. The model fitness was done using deviance in which the final model (Model III) was best fitted model since it had the lowest deviance (7168) (Table 3).

**Table 3:** Multilevel multivariable Analysis of Factors Associated with delayed initiation of ANC in Gambia

Individual and community-level variables	Models			
	Null model	Model I	Model II	Model III
	AOR (95%CI)	AOR (95%CI)	AOR (95%CI)	AOR (95%CI)
**Age**				
15–24		1		1
25–34		0.79 (0.68,0.91)		0.77(0.67, 0.89) **
35–49		0.79 (0.67,0.93)		0.77(0.65, 0.90) **
**Ethnicity**				
Mandinka/Jahanka		1		1
Wollof		1.23(1.01,1.59)		1.40(1.12, 1.75) **
Fula/TUkulur/Lorobo		0.92(0.76,1.11)		0.92 (0.77, 1.11)
Sarahule		0.75(0.55,1.01)		0.88 (0.66, 1.17)
Non-Gambia		1.22(0.99,1.52)		1.12 (0.90, 1.39)
Others		1.14(0.90,1.44)		1.00 (0.79, 1.26)
**Women Education**				
No education		1		1
Primary		0.97(0.83,1.14)		0.95 (0.81, 1.11)
Secondary		1.13(0.96,1.32)		1.07 (0.91, 1.25)
Higher		0.89(0.62,1.28)		0.81 (0.57, 1.16)
**Wealth index**				
Poor		1		1
Middle		1.31(1.10,1.56)		1.00 (0.83, 1.20)
Rich		1.36(1.13,1.64)		0.82 (0.67, 1.02)
**Had health insurance**				
Yes		1		1
No		1.73(1.11,2.71)		1.78 (1.14, 2.76) *
**Still Birth**				
No		1		1
Yes		1.41 (0.92,2.15)		1.40 (0.91, 2.14)
**Planned pregnancy**				
Yes		1		1
No		1.64 (1.41,1.90)		1.59 (1.37, 1.84) **
**CS History**				
Yes		1		1
No		1.40(1.02, 1.94)		1.50 (1.09, 2.07) *
Community level factors				
**Residency**				
Rural			1	1
Urban			0.65(0.52,0.81)	0.59(0.47, 0.75) **
**Local Government Area**				
Banjul			1	1
Kanifing			1.07(0.76,1.49)	1.17 (0.82, 1.67)
Brikama			1.60(1.16,2.21)	1.44 (1.03, 2.03) *
Mansakonko			0.82(0.55,1.22)	0.77(0.51, 1.18)
Kerewan			0.53(0.36,0.77)	0.43(0.29, 0.64) **
Kuntaur			0.65(0.44,0.97)	0.52 (0.34, 0.79) **
Janjanbureh			0.84(0.57,1.24)	0.75(0.50, 1.13)
Basse			0.63(0.44,0.91**)**	0.58(0.39, 0.85) **

### Determinants of late ANC initiation among reproductive age women in Gambia, GDHS 2019-20

On models, II and III bivariable analysis was done to identify candidate variables for multivariable analysis using p-value < 0.20. Therefore, women’s age, ethnicity, education, had health insurance, ANC, history of stillbirth, planned pregnancy, and CS history from the individual level variables and also residency and LGA from the community level variable were significant. In Model IV multivariable multilevel logistic regression model was done by incorporating both individual and community-level variables. The following variables were insignificant at a p-value < 0.05: women age, residency, LGA, ethnicity, had health insurance, ANC, planned pregnancy, and CS history. The odds of late ANC initiation among women age 25–34 and 35–49 decreased by 23% as compared to age group 15–24 (AOR = 0.77;95%CI: 0.67,0.89), and (AOR = 0.77;95%CI: 0.65,0.90) respectively. The odds of late ANC initiation among women from Wollof ethnicity increased by 40% as compared to women from Mandinka/Jahanka ethnicity (AOR = 1.40; 95% CI: 1.12, 1.75). The odds of late ANC initiation among women who had no health insurance coverage increased by 78% as compared to women who had health insurance coverage (AOR = 1.78; 95% CI: 1.14,1.76). The odds of late ANC initiation among women who had less than eight ANC visits were 18.26 times (AOR = 18.26, 95% CI 10.97,30.39) higher than women who had eight and more ANC visits. The odds of late ANC initiation among women who did not plan their last pregnancy increased by 59% as compared to women who planned their pregnancy (AOR = 1.59; 95% CI: 1.37,1.84). The odds of late ANC initiation among women who had no history of CS increased by 50% as compared to women who had had no history of CS (AOR = 1.50; 95% CI: 1.09,2.07). The odds of late ANC initiation among women rural residents decreased by 41% compared to women urban residents (AOR = 0.59; 95%CI: 0.47, 0.75). The odds of late ANC initiation among women residing in Brikama LGA increased by 44% as compared to women residing in Banjul LGA (AOR = 1.44; 95% CI:1.03, 2.03). The odds of late ANC initiation among women residing in Kerewan LGA decreased by 57% as compared to women residing in Banjul LGA (AOR = 0.43; 95% CI:0.29, 0.64). The odds of late ANC initiation among women residing in Kuntaur LGA decreased by 48% as compared to women residing in Banjul LGA (AOR = 0.52; 95% CI:0.34, 0.79). The odds of late ANC initiation among women residing in Basse LGA decreased by 42% as compared to women residing in Banjul LGA (AOR = 0.58; 95% CI:0.39, 0.85) (Table 4).

**Table 4:** Multilevel random effect analysis and model fitness on delayed ANC initiation in Gambia

**Random effects**				
**Community variance (SE)**	0.444 (0.062)	0.375 (0.060)	0.179(0.035)	0.168(0.035)
**ICC%**	11.90%	10.24%	5.17%	4.86%
**PCV%**	1	15.54%	59.68%	62.16%
**MOR**	2.17	2.04	1.63	1.61%
**Model comparison**				
**LR test vs. logistic model**	267.97**	168.35**	78.11**	62.88**
**LLR**	-3830.10	-3652.40	-3763.31	-3584.04
**Deviance**	7660.20	7304.80	7526.62	7168.08
**AIC**	7664.21	7342.79	7546.61	7222.08
**BIC**	7677.51	7469.17	7613.13	7401.66

## Discussion

Timely initiation of ANC was essential in a low-income country like Gambia because the outcomes for maternal and child health can be improved by timely introduction and sustained attendance at ANC programs. The objective of this study was estimating the magnitude and its predictors of delayed initiation of ANC among pregnant women in Gambia. The finding of this study revealed that the magnitude of delayed initiation ANC was 57% with a 95% Confidence Interval (CI) (56%; 59%) which is lower than studies conducted in South Africa [44], West Africa [54] and Ethiopia [55]. This implies that a significant proportion of expectant women begin their first ANC booking at the recommended gestational age. Early first ANC initiation offers a chance to collect baseline information on the mother’s general health and the fetus [37, 38]. The pregnant woman and fetus benefit from supplementing with iron and folic acid early in the first gestational age [11, 34, 56, 57]. Our finding indicated that women without health insurance have greater odds of delayed initiation of ANC compared with insured women consistent with previous studies reported elsewhere [58–60]. Since health insurance offers adequate protection from catastrophic costs and is linked to other socioeconomic factors like wealth and schooling, which are known to influence the onset of ANC early in life. Also, the finding of our study indicated that unplanned pregnancy have the higher odds of delayed initiation of ANC as compared to planed pregnancy consistent with other findings published in South Africa [44] and Ethiopia [31, 32, 50, 56]. Since unplanned pregnancies may cause people to consider abortion or deny they are pregnant, both of which may postpone the initiation of first ANC services [61, 62]. Since unplanned pregnancies are also associated with societal and cultural factors that affect health-seeking behaviours [61–63]. In addition, the finding of this study revealed that pregnant women reside in rural areas have higher odds of late initiation of ANC service consistent with previous studies conducted in Ethiopia [32, 35, 64] and Bhutan [65]. This is because women who live in rural areas frequently have poor economic status, limited media exposure, and limited access to healthcare, all of which limit how effectively ANC services are presented [66, 67]. While women who live in urban areas, experienced family members and friends, access to media, internet, and healthcare workers all played crucial role in providing information and guidance about the necessities of ANC. Additionally, pregnant women with older age have higher odds of late ANC presentation in line with earlier studies [66–69]. Since this is the fact that old women have multiple tasks and able to deny the necessity of early ANC visit in their as well as child health care service. Moreover, the finding of this study indicated that women were experienced previous caesarean delivery have higher odds of late ANC visit as compared to women delivered in vaginally.

### Strength and limitation of the study

The main strength of this study was that it used a nationally representative data with large sample size. The other strength was that we employed an advanced and appropriate statistical approach (multilevel analysis) to accommodate the hierarchical nature of the data. However, this study had limitations in that the GDHS survey is relied on respondents’ self-report and might have the possibility of recall bias because respondents/mothers were asked to remember things happened in the past. Again, this study only generates associations between delayed first ANC booking and some important individual-level and community-level factors that is limited in its design to establish causality between the outcome of interest and these important independent variables.

## Conclusion

Despite known benefits of early antenatal care initiation, the finding of this study revealed that the rate of late ANC booking is still high in Gambia. Unplanned pregnancy, residence, Health insurance, history of CS delivery, and age were significantly associated with delayed first ANC initiation.

### Recommendation

Health promotion initiatives that inform women about the necessity of early ANC booking, particularly those who lack or have little formal education, live in rural areas, or are older, should help to raise their level of knowledge about the advantages of doing so. The government and minister of Gambia should also inform the population about the value of health insurance in order to promote the use of maternal health services.

## Data Availability

The survey datasets used in this study was based on publicly available dataset that is freely available online with no participant’s identity from http://www.dhsprogram.com/data/available-datasets.cfm. Approval was sought from MEASURE DHS/ICF International and permission was granted for this use.

## Abbreviations

ANC: Antenatal Care
AOR: Adjusted Odds Ratio
CI: Confidence Interval
GDHS: Gambian demographic and Health Survey
ICC: Intra-class Correlation Coefficient
LLR: Log-likelihood Ratio
LR: Likelihood Ratio
MOR: Median Odds Ratio
SSA: Sub-Saharan Africa
WHO: World Health Organization.
